# Distribution of two invasive mosquito species in Slovenia in 2013

**DOI:** 10.1186/1756-3305-7-S1-P9

**Published:** 2014-04-01

**Authors:** K Kalan, VE Buzan, V Ivović

**Affiliations:** 1 Institute for Biodiversity studies, Science and research centre, University of Primorska, Koper, Slovenia; 2 Faculty of Mathematics, Natural Sciences and Information Technologies, University of Primorska, Koper, Slovenia

## 

In the recent years there has been growing interest in the establishment and spread of invasive mosquito species in Europe. Apart from the most dispersed, *Aedes albopictus*, there are five other alien aedine mosquito species spreading in Europe. Their spreading represents a considerable threat for public health as they can serve as vectors of mosquito-borne diseases.

A survey in whole Slovenia was performed in 2013 to determine the distribution of invasive mosquitoes. The study was based on the search for invasive mosquito larvae in artificial water containers at the cemeteries, around human dwellings and in used tires at vulcanizing companies. Additionally, a call for citizens to report any nuisance from "unusual" mosquitoes was published in local media.

With the study we have revealed the presence of two invasive mosquito species in Slovenia, *Ae*. *albopictus *and *Ae*. *japonicus*. Altogether we caught 494 larvae of *Ae*. *albopictus *and 1318 larvae of *Ae*. *japonicus*. The results show that *Ae*. *albopictus *is present mostly in Southwestern and central part of the country, with some isolated locations in other parts of Slovenia. Nevertheless, *Ae*. *japonicus *was found in a large part of the investigated area. This species was previously collected from a small area near the Austrian border in 2011, and in only two years it has colonized the majority of Northeastern part of Slovenia.

In Slovenia no systematic mosquito surveillance or control is currently done. There are only some minor projects, which are carried out by local institutions at a regional level. *Ae. albopictus *and *Ae. japonicus *are spreading fast and they both present high nuisance for the humans, as well as a public health risk since transmitting vector borne diseases. Therefore an effective national monitoring program of invasive mosquito species is highly needed. With well planned, long term strategy we will be able to detect and control their occurrence, monitor their pathogens and prevent the establishment of new foci of mosquito-borne diseases.

